# Seasonal variation in defense behavior in European and *scutellata*-hybrid honey bees (*Apis mellifera*) in Southern California

**DOI:** 10.1038/s41598-023-38153-2

**Published:** 2023-08-07

**Authors:** Daniela Zarate, Brandon Mukogawa, Joshua Kohn, James C. Nieh

**Affiliations:** Department of Ecology, Behavior, and Evolution, School of Biological Sciences, University of California San Diego, 9500 Gilman Dr., MC 0116, La Jolla, CA 92093-0116 USA

**Keywords:** Behavioural ecology, Behavioural genetics

## Abstract

Nest defense in the honey bee (*Apis mellifera*) is a complex collective behavior modulated by various interacting social, environmental, and genetic factors. *Scutellata-*hybrid (“Africanized”) honey bees are usually considered to be far more defensive than European honey bees which are therefore preferred for commercial and hobbyist beekeeping. In the most recent zone of *scutellata* hybridization, the southern USA, the degree to which this defensiveness differs among current strains, and the extent to which defensiveness varies across a season has not been measured. We quantified the levels of *A. m. scutellata* ancestry in colonies and conducted a seasonal assessment (May through November) of colony nest defensiveness in feral *scutellata-*hybrid and a popular lineage of European honey bee commonly used in managed environments (sold as *A. mellifera ligustica*) hives at two apiaries in Southern California. Standard measures of defensiveness were low in both *scutellata-*hybrid and European colonies during May. Defensiveness increased during the later months of the study in *scutellata*-hybrid colonies. Most measures of defensiveness did not increase in managed colonies. Defensiveness in the *scutellata-*hybrids appears lower than what has been previously documented in Brazil and Mexico, possibly due to their lower proportion of *A. m. scutellata* ancestry.

## Introduction

Multiple social insects possess an impressive capacity for nest defense—mobilizing hundreds to thousands of individuals through a complex cascade of information transfer, physiological response, and behavioral modification^1^. The study of such complex colony-level behavior poses special challenges because this behavior is determined by various social, genetic, and environmental factors interacting in ways that are not easily teased apart (reviewed in^2^). In honey bees, there is high interest in assessing the relative contributions of these variables to defensive behavior due to the intensive handling of colonies inherent in beekeeping, which runs the gamut from small-scale hobbyist practices to commercial-level apiculture and pollination.

There are marked differences in nest defense behavior amongst the more than two dozen recognized subspecies of the western honey bee. These subspecies fall into several broader biogeographic clades: Africa (A), Western Europe (M), Eastern Europe (C), Middle East (O), and Arabian Peninsula and Eastern Africa (Y) (reviewed in^3^). Defensiveness varies greatly within and across clades^4^. Certain subspecies are characterized by a low level of defense (e.g. *A. m. ligustica*, the Italian honey bee and *A. m. monticola,* the Ethiopian highlands honey bee) and others for their propensity to collectively sting and harass in response to a disturbance of their hive (e.g. *A. m. scutellata,* a widespread African subspecies, and the Syrian honey bee, *A. m. syriaca*)^4,5^. In the United States, one of the most widely used honey bee strains for both commercial-scale services and backyard beekeeping due to its low defensiveness, reduced proclivity to swarm, and robust honey production is the one sold as the Italian honey bee (*A. m. ligustica*)^6,7^. However, genetic analysis of honey bees used commercially show them to be hybrids whose genomes are made up nearly entirely of a mixture of C and M lineages^8^. In addition, honey bees from the Western European (M) and Middle Eastern (O) lineages have also been introduced to the New World, and often hybridize with honey bees in both feral and managed populations^6,9^. We will refer to these commercial hybrids whose genomes are dominated by European lineages as European Honey Bees (EHB).

The introduction of *A. m. scutellata* honey bees to Sao Paulo, Brazil in 1956—and their subsequent escape a year later—brought this African subspecies into contact with the previously introduced subspecies (EHB), with which they hybridized^10^. Because of negative, and in some cases, inaccurate connotations we avoid the term “Africanized” and adopt the term “*scutellata*-hybrid” first suggested by Calfee et al*.*^11,12^. Heightened defensiveness is hypothesized to be one of the principal factors underlying the widespread success of *scutellata-*hybrid honey bees following their introduction to the American continents^13^. Additionally, *scutellata-*hybrids exhibit higher rates of absconding and lower honey production, behaviors deemed undesirable for apiculture, but that potentially helped *scutellata-*hybrids rapidly expand their range^13^. The increase in human fatalities from honey bee stings following the arrival of these hybrids in Mexico (480 fatalities out of more than 5000 individual cases) caused widespread public fear, leading them to be called “killer” bees^14^. As a result, the number of beekeepers in Mexico decreased^15^. However, the initial reluctance to work with *scutellata-*hybrids was short-lived and this hybrid is now widely used across Mexico, Central America, and South America—an acclimatization made possible through improved management practices and the ability of this hybrid to survive and thrive in these regions^16^. However, in the United States, working with *scutellata-*hybrids is still largely avoided and genetic intervention (i.e. requeening) is encouraged to prevent interbreeding between feral *scutellata-*hybrids and managed honey bees in regions where *scutellata-*hybrids have become established (e.g. the southwestern United States)^13^.

Standardized assays of honey bee defensive behavior—measured on individual, small-group, and colony-level scales (reviewed in^17^) have demonstrated that *scutellata-*hybrids are usually more defensive than European honey bees (e.g. *A. m. carnica* and *A. m. ligustica*)^13^. In comparison to EHB, *scutellata*-hybrids exhibit lower response thresholds to a disturbance or to alarm pheromone (isopentyl acetate, IPA), recruit more nestmates, and, ultimately, deploy a higher proportion of colony workers as defenders^18–22^. *Scutellata*-hybrid colonies assessed in Brazil stung a target up to 8.5 × more than EHB colonies within the first 90 s of observation and pursued the observer up to 7 × greater distances^18,19^ However, in a similar study conducted in Mexico, EHB colonies often failed to pursue a target at all^23^. In co-fostered *scutellata*-hybrid/EHB colonies, the hybrids comprised the majority of the defenders that responded in the first 10 s after a disturbance as well as nearly 70% of total pursuing honey bees^23^. In such mixed *scutellata*-hybrid/EHB colonies, the presence of *scutellata*-hybrids also caused European workers to act more defensively^23,24^.

While elevated defensive behavior is strongly tied to African ancestry, the genetics underlying this complex behavior remain unclear. Nest defense is heritable and displays a paternal effect: defensiveness is higher if *scutellata*-hybrid drones are mated with EHB queens than the reverse^20,21,25–27^. Quantitative trait loci (QTL) have been identified that correlate with increased measures of defensiveness (e.g. tendency to sting) at both the colony and individual levels^28,29^. In a separate study, backcrossed *scutellata*-hybrids carrying the African allele for a marker linked to the QTL for stinging behavior (*sting-*1) responded more rapidly to a disturbance and were more likely to sting^21^.

However, not all *scutellata*-hybrids display heightened levels of defensiveness. Gentle *scutellata*-hybrids are found in Puerto Rico^30–33^. These gentle hybrids have somewhat lower *A. m. scutellata* total genomic content than *scutellata*-hybrids in Central and South America (~ 50–70% in Puerto Rico in comparison to ~ 80% where defensive behavior of *scutellata*-hybrids has been measured in Brazil and Mexico^30,31^. However, in a region strongly associated with colony-level defensiveness (on LG 07), European ancestry is significantly overrepresented^30^.

In addition to genetic factors, environmental and social factors can also modulate defensiveness. Larger colonies can show increased defensiveness and infestation with *Varroa* parasites can correlate with increased defensiveness and a lower threshold for stinging^34–36^. Immune challenges are associated with decreased defensiveness^37^. Climatic factors such as increased temperature, humidity, and pressure can also affect colony defense^38^. In addition, resource limitation can heighten honey bee robbing behavior and has been shown to increase defensiveness in guards on both behavioral and transcriptomic levels^39,40^. Colony defensiveness may also increase during reproductive swarming or as a response to predation^30^. In the context of management, defensiveness has been reported as decreasing after repeated manipulation by a beekeeper, although this has not been quantitatively studied.

Honey bee defense behavior is also of interest because it influences management policies and practices. In regions such as Southern California, where *scutellata*-hybrids arrived in 1994 and now dominate the very large feral honey bee population, frequent requeening with European varieties is mandated to mitigate the genetically-based defensiveness of *scutellata*-hybrids^41–43^. While many beekeepers follow these practices, the extent to which such requeening maintains genetic purity and gentleness in an area with abundant *scutellata*-hybrids is unclear. Currently, there has been no quantitative assessment of defensiveness in *scutellata*-hybrid honey bees in Southern California, or anywhere in the United States, and there are no studies of how defensiveness may vary across a season. We therefore conducted genomic profiling and measured colony-level defensiveness over time in feral *scutellata*-hybrids and, separately, in managed honey bees. We hypothesized that *scutellata*-hybrid honey bees would be more defensive when they had significantly greater *scutellata-*ancestry, when their colonies were larger, and that defensiveness would also shift throughout the year as colony sizes increased.

## Materials and methods

### Honey bee colonies and apiaries

All honey bee hives were kept in two apiaries in San Diego, California (U.S.A.). Managed EHB colonies were maintained at the Biological Field Station (BFS) on the University of California San Diego campus in La Jolla, CA and feral bees were kept at the Elliot Chaparral Reserve (ECR), a largely undisturbed coastal sage scrub habitat (Fig. S1). Although a common garden design with all colonies maintained in the same apiary is desirable to control for multiple environmental factors, we used separate apiaries for the following reasons. First, the presence of *scutellata*-hybrids with EHB colonies in the same apiary has been shown to increase the defensiveness of the EHB colonies^39,40^ We wished to independently measure the defensiveness of feral and managed colonies over time. Using separate apiaries prevented this interaction problem. In addition, for multiple years prior to this study, we hosted both *scutellata*-hybrids and EHB at the BFS apiary, not by design, but because our EHB colonies inevitably requeened themselves and, given the density of feral *scutellata*-hybrids throughout the region, these new queens often mated with *scutellata*-hybrid drones^42–44^. Over time, EHB colonies at the BFS often became notably more defensive. Using the standard black flag test (see below) to measure defensiveness, we found that nearly all colonies, including EHB (confirmed by the presence of marked EHB queens) had elevated levels of defensiveness, corroborating the observations of other researchers^39,40^. In addition, when we placed *scutellata*-hybrid and EHB colonies within the same apiary, we observed robbing in October and November. As a result, a high proportion of EHB colonies were robbed out and died, and their defensiveness could not be measured. Robbing also leads to increased defensiveness in an apiary^39^. The use of two separate apiaries therefore enabled us to conduct a study of defensiveness over an extended period of time. However, we recognize that multiple factors differed between these two sites and we therefore do not directly compare defensiveness between our *scutellata*-hybrid and EHB colonies. Instead, we focus on how defensiveness varied over an extended time period, the longest and most detailed assessment to date for studies of defensiveness by these two bee types.

Our managed colonies (BFS apiary) were inspected monthly and managed following standard beekeeping practices^45^. Managed colonies were founded as nucleus colonies purchased from northern California, outside of the current range of *scutellata*-hybrids. They were also annually requeened with Varroa Sensitive Hygiene (VSH) *A. m. ligustica* queens bred for gentleness and obtained from Wildflower Meadows, an apiary located in Vista, CA, with the most recent requeening occurring in July 2021. In each BFS colony, the presence of the same marked queen in every colony was confirmed during each monthly inspection, with the exception of colony #8 in which the marked queen was confirmed in August, but had been replaced by an unmarked queen by the November inspection. This unmarked queen may have mated with *scutellata*-hybrid drones, but the defensiveness exhibited by this colony in November was not markedly different from its defensiveness in August.

Any BFS colonies that naturally requeened had their queen removed and were requeened with these VSH queens. From August–March, managed colonies were provided with sucrose solution (50% w/w) ad libitum and protein patties (Ultra Bee High Protein Pollen Substitute, Mann Lake LTD). EHB colonies were also placed on hive stands that excluded ants.

In contrast, feral honey bee colonies (ECR apiary) were unmanaged and originated from feral swarms captured throughout San Diego County, likely to have ancestry admixed with *A. m. scutellata* (^43^ and see “Results” below). Feral colonies were not requeened nor treated for *Varroa* or any other diseases. No sucrose or protein supplements were ever provided to these colonies, and they did not have ant excluders.

At both apiaries, each colony was housed in a single standard 10-frame Langstroth hive. Ten colonies were chosen from each apiary (total *n* = 20), based on comparable sizes and similar honey stores and brood comb areas. When any of these colonies absconded or died, we chose another colony in order to maintain a sample size of 10 colonies evaluated per site each month. In total, 13 colonies across both sites (BFS, *n* = 7; ECR, *n* = 6) remained throughout the entirety of the study while all other colonies included in the study were only assessed for a portion of the study duration (ranging from 2–4 of the 5 months in which measurements were made).

### Defensiveness behavioral assays

Colony level behavioral assays were conducted on days with negligible to no cloud cover since weather can affect bee defensiveness^34^. Air temperatures were recorded at the beginning of assays on each day from temperature data loggers placed in the shade at each site (Hobologger©, Onset, USA). We conducted a modified version of the ratings assays described by Guzman-Novoa et al*.* and recorded six measures of colony defensiveness^46^. To standardize the scoring, only one researcher conducted the defensiveness tests (D. Zarate).

For each trial, the researcher wore sterile latex gloves over standard leather beekeeper gloves that were disposed of and replaced after each assay. Hives were opened, two puffs of smoke were applied to the tops of the frames, and two brood frames were removed and closely observed, simulating a hive inspection. The operator then ranked each colony on the tendency of honey bees to (1) fly off the comb, and (2) fly off and hit the operator’s veil. We ranked these measures on a scale of 1–5 with 5 being the most defensive and used a scoring rubric (Table S1). For example, for the metric of “Fly off Comb”, an estimate of 0–10 honey bees flying off and around the colony and observer merited a score of 1 whereas an estimate of 50 + honey bees resulted in a score of 5. The brood frames were then returned to the hive. We then (3) waved a black leather flag (6 cm × 6 cm and attached to 4 cm white binder clip that was cleaned to remove odors between trials) briskly over the brood frames at approximately ~ 1 wave/s for 15 s. The black flag was then deposited in a clear plastic bag and the number of stings deposited on it was counted at the conclusion of the trial. The top of the hive was replaced and then extent and intensity of honey bee pursuit was measured.

The observer retreated to pre-measured distances of (4) 25 m and then (5) 50 m from the focal colony. At each distance, the number of bees pursuing the observer was estimated and ranked on the same 1–5 scale with 5 being the highest pursuit level. The researcher then removed themselves from the trial area and waited until all honey bees had ceased pursuit before (6) counting the number of stings deposited on the latex gloves covering the leather gloves. In most cases, honey bees stung the gloves within the first few minutes of the assay when the operator was actively manipulating the colony and waving the black leather flag. Latex gloves were removed after each trial and leather gloves were subjected to a few puffs of smoke in order to dissipate any alarm pheromone that might have transferred to the leather.

To ensure that the defensiveness assays did not influence the behavior of surrounding hives, subsequent defense assays were conducted once all honey bees had ceased to pursue the observer. Colonies were assessed in the same order every day, and to limit the potential effects of defensiveness on nearby colonies, we based our order on testing one colony and then choosing a colony that was the furthest away from the previously tested colony. We also analyzed the effect of order on colony defensiveness. We assessed all colonies from both sites on each day measurements were made. Colony assays were repeated three times over three consecutive days spanning the late morning to early afternoon (10:00–15:00) and each defensiveness measure was averaged over these three days to obtain a more reliable estimate. Colony assays were conducted in 2021 in the months of May, July, August, September, October, and November.

### Honey bee colony size measurements

To assess the effect of colony size on measures of defensiveness, we measured the number of bees per colony each month, a few weeks before conducting each of the defensiveness assays. We counted bees using a modified version of the methodology described by Delaplane et al*.*^47^. Our method is based upon the Liebefeld method but also considered the variable bee densities on each frame side, instead of using a fixed estimate of 1000 workers per deep Langstroth frame^48^. We took photos of both sides of every frame containing bees using iPhones (XR and 13). Using GIMP 2.10 software, we overlaid a 5 cm × 7 cm grid on these photos to subdivide each bee frame into smaller grids. On each frame, we selected a 5 cm × 7 cm rectangle with a bee density that represented the density found in the majority of the grids and counted the number of individual bees in that cell. We then multiplied this value by the number of cells occupied by bees in the frame. We repeated this process for both sides of each frame and then summed these counts to calculate the number of workers per colony. To validate this method, we randomly selected some frames and counted all bees, and compared these counts to our estimates of bee numbers from the same frames. To ensure consistency between researchers counting bees, we randomly selected frame photos and had both researchers count bees on the same photos. We used linear regression to compare the counts made by the trainer and trainee. Trainees needed to obtain R^2^ ≥ 0.85 with the trainer before they were allowed to collect count data used in our analyses. We were not able to collect size data in July 2021. We also did not have size estimates for three colonies in October. We therefore ran our statistical models without size data for these colonies at these time points.

### Honey bee genomic sequencing

To assess the genetic composition of honey bee colonies from each apiary, we collected three honey bee workers from each colony (total n = 60). To ensure that these bees were from the hives being studied and not non-nestmates (e.g. robbers), we selected young bees (bees that had less wing wear and prominent white thoracic hairs) on brood combs. Honey bees from the ECR apiary were sampled in mid-November 2021, following the last defensiveness assay. Honey bees from the BFS apiary were sampled in late August, approximately 2 months after the last requeening (in early July 2021). Honey bees were euthanized and preserved in 100% ethanol at − 20 °C. We separately extracted DNA from the crushed head of each bee using the standard protocol of the Qiagen DNAeasy Blood and Tissue extraction kit (Catalog ID: 69504). The DNA was submitted for DNA KAPA library construction and whole-genome sequencing at the Institute for Genomic Medicine (IGM), UC San Diego. All 60 individuals were multiplexed and sequenced across three lanes of an Illumina NovaSeqS4 platform to produce 150-bp paired-end reads at ~ 20 × coverage.

### Assessing differences in nest defense by calendar month, colony size, and site

To assess the effect of colony size on defensiveness, we conducted a multivariate analysis of variance (MANOVA) for each month where we included all defensive measures as response variables and size as the predictor variable. Each site was analyzed separately. To assess differences in defensive behavior as a function of time and colony size, we applied a repeated measures mixed-effects model, again analyzing each site separately. For this analysis, we analyzed each defensive measure individually, designating it as a response variable. We included time, size, and any significant interactions between main effects in the model. We also included colony as a random effect as well as the interactions of time and colony, colony and size, and the interaction of time, colony, and size—designating these as random effects as well. We log-transformed the stings on gloves data based upon inspection of model residuals. Following standard practice, we first ran full models with all interactions and then ran the same model without non-significant interactions. Because the same defensiveness measures at any given time point were tested twice, once in the MANOVA and then in the mixed model repeated measures analysis, we applied the Dunn-Sidak correction and assess the results as significant if *p* < 0.0253. All analyses were performed using JMP v16.1, SAS Institute Inc, Cary, NC, USA.

To determine if the order of colony testing influenced colony defensiveness, we ran separate repeated measures models with each of the defensiveness behaviors as the response variable, order of sampling and Julian date as fixed effects, colony as a random effect, and all interactions. Interactions containing colony were random effects. This analysis was conducted separately for each site. We did not include colony size in this analysis because it was not significant in our prior analyses (see above). Order did not emerge as a significant predictor of any defensive measure at either site and so was excluded from all subsequent analyses (Table S2).

### Assessing the relationship between amount of African genomic ancestry and nest defense

To assess the extent to which amount of African ancestry predicted defensiveness, we ran a multivariate analysis of covariance (MANCOVA) with all defense metrics as response variables, percent *A. m. scutellata* ancestry as a model effect, and colony size as a covariate. We analyzed BFS and ECR separately as there was little variation in the percent of African ancestry in ECR. We used only the November behavioral data because ECR colonies were the most defensive at this time, based upon a majority of measurements. We also assessed the extent to which colony identity explained variation in African ancestry among the three bees sampled from each colony using an analysis of variance (ANOVA) for each site separately. All analyses were performed using JMP v16.1, SAS Institute Inc, Cary, NC, USA.

### Genomic admixture analysis

To create an ancestral reference panel, we leveraged two previously sequenced genomic honey bee datasets consisting of a combination of 99 whole honey bee genomes, representing the four major continental honey bee clades (A, M, C, O) and seven different honey bee subspecies (29 genomes from Harpur et al*.* and 70 genomes from Wallberg et al*.*)^49,50^. Wallberg et al*.* generated their genomes via whole genome sequencing on a SOLiD 5500xl platform (75-bp reads; average coverage 4.4 × ± 1.5 × per individual). Harpur et al*.* sequenced their genomes using Illumina Hi-Seq (50-bp reads; average coverage of 38 × per individual). In total, across both sequencing projects, we assembled a reference panel consisting of 21 genomes of the African (A) subspecies *Apis mellifera scutellata*; 29 genomes of two Western European (M) subspecies [*Apis mellifera mellifera* (*n* = 14) and *Apis mellifera iberiensis* (*n* = 15)]; 29 genomes of two Eastern European (C) subspecies [*Apis mellifera carnica* (n = 19) and *Apis mellifera ligustica* (*n* = 10)]; and 20 genomes of two Middle Eastern (O) subspecies [*Apis mellifera anatoliaca* (*n* = 10) and *Apis mellifera syriaca* (*n* = 10)]. Raw reads from these datasets were obtained from NCBI [Wallberg et al*.*: Project ID: PRJNA236426; and Harpur et al*.* (accession no. SRP029219)]. Raw reads generated from sequencing and those downloaded from NCBI were trimmed and filtered for quality and length using the program POPOOLATION v1.2.2 (--quality-threshold 25 --min-length 40)^51^. While POPOOLATION is designed for pooled data, it can filter unpooled data such as ours. We then aligned reads to the most recent honey bee reference genome (Amel_HAv3.1—assembled by Wallberg et al*.*^52^) using BWA v0.7.12 (bwa mem algorithm under default parameters)^53^. Mapped reads were then sorted and filtered for mapping quality (alignments with mapping score of q < 20 were discarded) using the program SAMTOOLS^54^. In order to mitigate PCR amplification bias introduced during sequencing, duplicate reads were removed using the program PICARD (MarkDuplicates)^55^.

Following duplicate read removal, we used the program ANGSD v0.930^56^ to estimate genotype likelihoods using the SAMTOOLS model (-GL 1) across all references and sample genomes (n = 159). We performed BEAGLE haplotype imputation (-doGLF 2) by inferring major and minor alleles (-doMajorMinor), estimated allele frequencies (-doMaf 1), tested for polymorphic sites (SNPs) and kept only those with a likelihood ratio test of p < 1e−6 (-SNP_pval 1e−6). We additionally excluded genotypes with base quality below 20 (-minQ 20) and excluded alignments with mapping quality below 40 (-minMapQ 40). We also removed triallelic sites, confidently identified with a likelihood ratio test of p < 0.001 (-rmTriallelic 0.001). The resulting SNP dataset was then thinned for linkage disequilibrium by keeping 1 out of every 100 markers. Because honey bees have relatively high levels of recombination and given that linkage disequilibrium has been shown to decay rapidly, 1500 bp spacing in between markers is sufficient to have r^2^ < 0.1 in the large continental clades (A, C, M), and 1000 bp spacing achieves r^2^ < 0.2 in between any subspecies^49^.

We then used the program NGSADMIX to estimate individual ancestry. NGSADMIX is an ancestry estimation algorithm based on genotype likelihoods rather than genotypes, allowing the program to take sequencing uncertainty into account during ancestry inference and allowing for a robust estimation of ancestry in spite of low coverage data^57^. During ancestry estimation we allowed only sites with a minor allele frequency greater than 5% (-minMaf 0.05) and informative in at least 150 of the 159 individuals (94% of individuals) (-minInd 150). The final SNP dataset used to estimate ancestry consisted of 201,975 SNPs. We did not perform a supervised clustering analysis by designating which individuals were the ancestral population.

Using these parameters, we conducted five separate NGSADMIX runs, specifying a different number of ancestral populations ranging from 2 to 6 (K = 2–6). We chose to focus on K = 4 as the number of assumed clusters because several studies have shown that variation in honey bee ancestry is best subdivided into the four major continental groups (A, M, C, O)^49,58^. In addition, Nelson et al*.*^58^ used the program ADMIXTURE to assess genomic ancestry in *scutellata*-hybrid honey bees in Brazil and showed that the CV-error did support K = 4 as the optimal K value. Admixture results were plotted using R^59^. All bioinformatic scripts are publicly available on the author’s GitHub: https://github.com/danielazarate/Honey-Bee-Genomic-Admixture.

## Results

### Nest defense behavior modulated by size and calendar month

**Figure 1:**
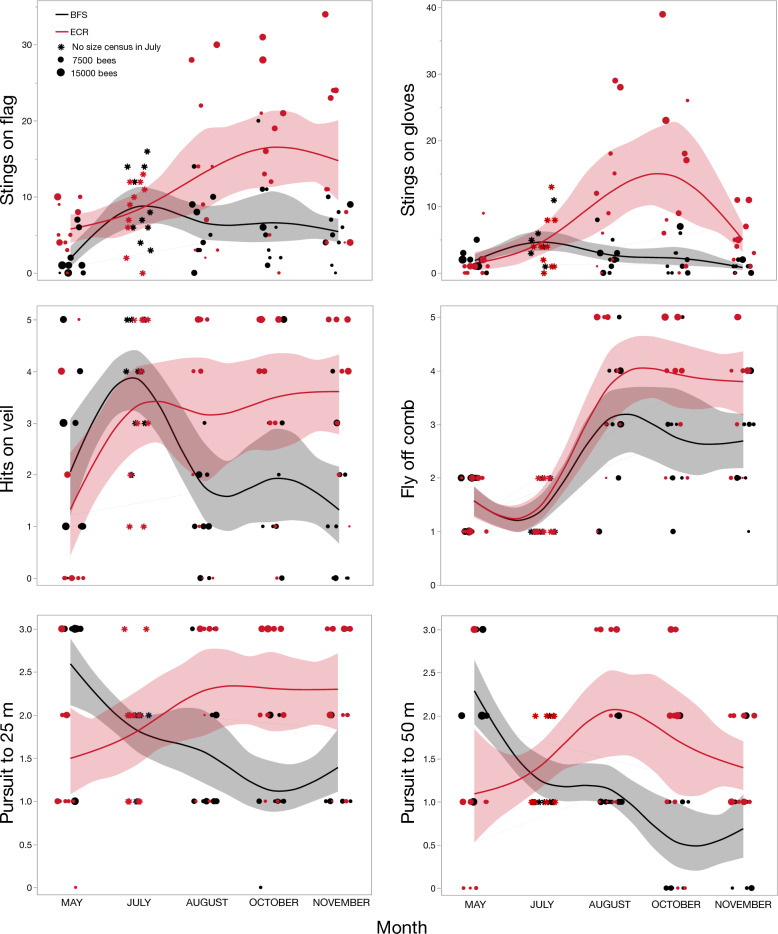
Honey bee colony defensive measures. The stinging measures are continuous variables while all other measures are ordinal variables, ranked on a scale of 1 to 5 with 1 being the least defensive and 5 the most defensive. Plots show spline lines and 95% confidence intervals calculated based upon a model including month and colony size (indicated by dot size).

Colony sizes are reported in Table S3. Colony size was not a predictor of defensiveness (all six measures) as assessed by MANOVA for each site and month separately, using an alpha level significance threshold of *p* < 0.0253 (Table S4). When each measure of defensiveness was analyzed separately across all months; colony size, time, and the interaction of these two variables significantly predicted some measures of defensiveness (*p* < 0.0253) (Table 1, Fig. 1).

**Table 1:** Repeated measured mixed-effects model assessing the effects of colony size and time on each defensive measure with each site analyzed separately. We included time, size, and colony and any significant interactions between main effects in the model. Colony and any interactions containing colony were designated as random effects. Stings on gloves were log transformed to improve model residuals. The analyses included only colonies for which we had data for the entire duration of the study (BFS: *n* = 7, ECR: *n* = 6). For October at our ECR site, we were unable to use MANOVA to analyze the effect of colony size on defensive behavior due to the small number of colonies (*n* = 5) for which we had size estimates. Non-significant interaction effects were excluded from the final models reported. We applied a Dunn-Sidak correction and assess the results as significant if *p* < 0.0253, marked with an asterisk (*).

Site	Defensive measure	Source	Nparm	DF	DFDen	F ratio	Prob > F
BFS	Stings on flag	Month	3	3	35	3.06	0.041
		Size	1	1	0.16	< 0.002	0.985
	Stings on gloves	Month	3	3	35.0	4.96	0.006*
		Size	1	1	35.0	0.44	0.509
	Fly off comb	Month	3	3	26.8	6.26	0.002*
		Size	1	1	11.2	1.16	0.303
	Hit veil	Month	3	3	35.0	0.68	0.569
		Size	1	1	8.72	2.98	0.119
	Pursuit 25 m	Month	3	3	23.8	9.24	0.0003*
		Size	1	1	9.41	1.15	0.311
	Pursuit 50 m	Month	3	3	29.8	20.8	< 0.0001*
		Size	1	1	12.5	2.17	0.165
ECR	Stings on flag	Month	3	3	20.9	2.33	0.052
		Size	1	1	5.17	14.8	0.0004*
	Stings on gloves	Month	3	3	19.3	26.2	< 0.0001*
		Size	1	1	3.3	10.2	0.044
		Month × Size	3	3	12.8	15.8	0.0001*
	Fly off comb	Month	3	3	15.8	15.6	< 0.0001*
		Size	1	1	8.7	11.2	0.009*
	Hit veil	Month	3	3	13.3	6.69	0.006*
		Size	1	1	5.02	7.21	0.043
	Pursuit 25 m	Month	3	3	18.6	9.70	0.0004*
		Size	1	1	7.35	6.17	0.0405
	Pursuit 50 m	Month	3	1	3.35	16.0	0.018*
		Size	3	1	7.13	5.67	0.0482

At the BFS site, month significantly predicted stings on gloves (F_3,25_ = 3.06, *p* < 0.01), the tendency for bees to fly off the comb (F_3,27_ = 6.26, *p* < 0.01), and pursuit to 25 m (F_3,24_ = 9.24, *p* < 0.001) and 50 m (F_3,30_ = 20.8, *p* < 0.001). For three of these measures (stings on gloves and both pursuit measures), defensiveness significantly decreased over time, but flying off the comb significantly increased over time (Fig. 1). However, colony size and the interaction of size and time were not significant predictors of any measure of defensiveness (*p* > 0.0253).

In contrast, at our ECR site, colony size significantly predicted stings on flag (F_1,5_ = 14.8, *p* < 0.001) and the tendency for bees to fly off the comb (F_3,16_ = 15.6, *p* < 0.01). At the ECR site, five measures of defensiveness increased over time: month predicted stings on gloves (F_3,19_ = 26.2, *p* < 0.001), the tendency for bees to fly off the comb and hit the operator’s veil (F_3,13_ = 6.69, *p* < 0.01), and pursuit to 25 (F_3,19_ = 9.70, *p* < 0.001) and 50 m (F_1,4_ = 16.0, *p* < 0.0253). For stings on gloves, the interaction of colony size × month was significant (F_3,13_ = 15.8, *p* < 0.001) (Fig. 1). Colony and any interactions with colony did not emerge as significant variables and so were removed from the final models.

### Genomic admixture estimates

**Figure 2:**
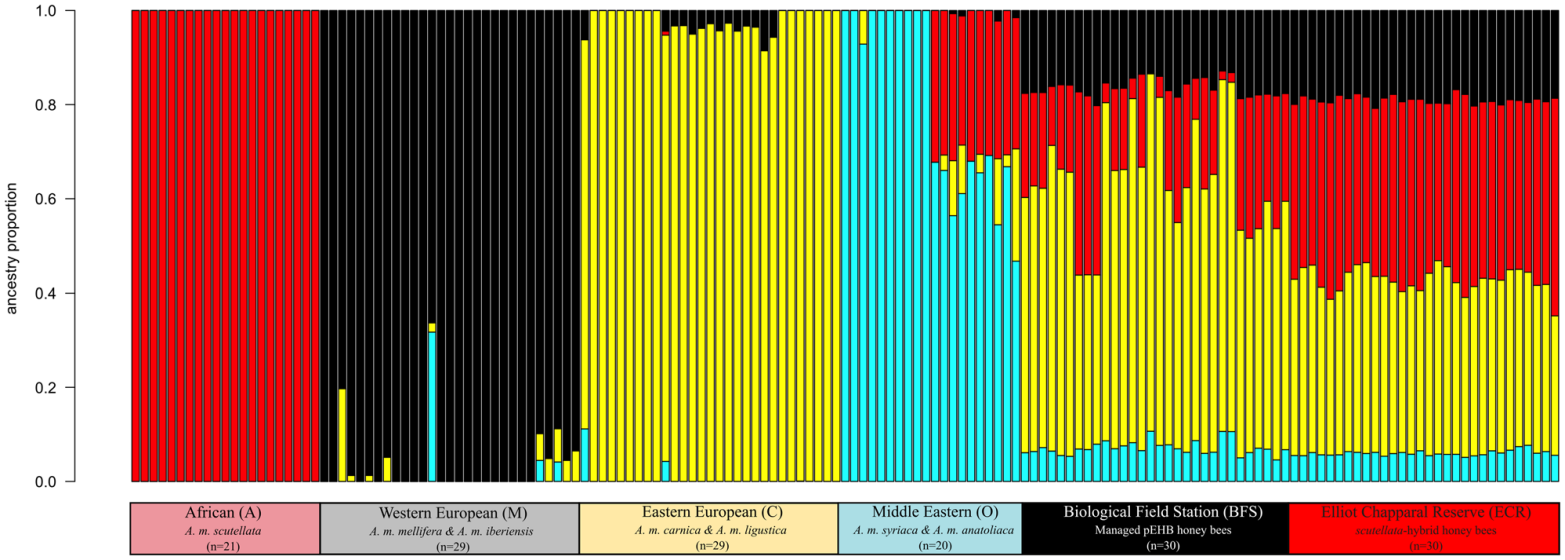
NGSadmix barplot of ancestry (K = 4). Each vertical bar represents one honey bee genome and colors represent the estimated proportion of ancestry derived from each assumed ancestral genetic cluster. Starting from the left of the figure and beginning with the African clade, we list reference genomes belonging to the four major evolutionary lineages of *Apis mellifera* (A, M, C, O). Following these, we list 60 admixed honey bee genomes, half collected from feral hives and half collected from managed hives present in San Diego, CA, USA.

Colonies at ECR and BFS differed significantly in their amounts of *A. m. scutellata* ancestry (*p* < 0.001). BFS colonies were on average 19% African (A Clade), 57% Eastern European (C Clade), 16% Western European (M Clade), and 7% Middle Eastern (O Clade) (Fig. 2, Table 2A). In contrast, ECR colonies exhibited higher *A. m. scutellata* ancestry on average (38%), followed by Eastern European (37%), Western European (19%) and Middle Eastern (6%) (Fig. 2, Table 2A). All feral colonies therefore displayed a percentage of *A. m. scutellata* ancestry typical of feral bees previously sampled in Southern California^11,43^.

**Table 2:** Genomic composition and its lack of association with defensiveness. (A) Genomic composition (mean ± se) of honey bee workers sampled from each colony by site and (B) multivariate analysis of covariance (MANCOVA) of honey bee defensiveness measures as a function of African ancestry for each site.

	BFS	ECR			
(A) Genomic composition					
African (A)	0.192 ± 0.019	0.382 ± 0.004			
Western European (M)	0.165 ± 0.004	0.190 ± 0.001			
Eastern European (C)	0.572 ± 0.022	0.369 ± 0.004			
Middle Eastern (O)	0.0718 ± 0.003	0.060 ± 0.001			

Site	Effect	*F*	NumDF	DenDF	*P*-value
(B) Defensiveness as a function of African ancestry in November					
BFS	Size	0.86	6	2	0.627
	African Ancestry	1.05	6	2	0.564
ECR	Size	2.47	6	2	0.316
	African Ancestry	1.86	6	2	0.390

There was considerable variation in *A. m. scutellata* ancestry between colonies in the BFS apiary: average *A. m. scutellata* content ranged between 7 and 38%. We will hereafter refer to these colonies as predominantly EHB (pEHB). Only one BFS colony had levels of *A. m. scutellata* ancestry that overlapped with estimates for ECR colonies, which ranged from 35 to 41% (colony averages). Colony identity explained ~ 75% of variation in *A. m. scutellata* ancestry in separate analyses of ECR and BFS apiaries (*p* < 0.01), indicating relatively little variation in ancestry among bees sampled from the same colony compared to between-colony variation (Table S5).

The amount of *A. m. scutellata* ancestry was not a significant predictor of nest defensiveness, as assessed by MANCOVA of all six behavioral measures with colony size and percentage of African ancestry as covariates (*p* ≥ 0.39; Table 2B). This was true at both the BFS (*p* > 0.56) and ECR (*p* ≥ 0.32), analyzed separately, for the month of November (Table 2B).

## Discussion

Overall, defensive behaviors of our *scutellata*-hybrid and managed colonies (pEHB) significantly changed over time, and thus judging a colony’s ancestry based on its behavior at any given moment is unreliable. The defensiveness of *scutellata*-hybrid colonies at ECR generally increased over time and was affected more by time than by colony size (Fig. 1, Table 1). At ECR, only two measures (stings on flag, fly off comb) showed a significant effect of colony size alone, and only one showed a significant interaction between month and size (stings on gloves) whereas four measures (fly off comb, hit veil, and pursuit to 25 and 50 m) showed a significant effect of month alone. For these measures, larger colonies showed increased measures of defensiveness. Even in the months of highest defensiveness, our *scutellata*-hybrid colonies were evidently not as defensive as the *scutellata*-hybrid colonies assessed with similar tests in Mexico, Brazil or Venezuela^18–25,24^. The defensiveness of *scutellata*-hybrid colonies, as measured by sting rate (the number of stings accumulating on an object over time) has been quantified in several studies that have primarily been conducted in Mexico and Central and South America (Table S6). Sting rates by *scutellata*-hybrid colonies are variable: 46.7 stings/min^60^, 61.2 stings/min^18^, 110 stings/min^25^, 125 stings/min^21^, 128.9 stings/min^20^, 145.2 stings/min^24^, 164 stings/min^61^, 188 stings/min^23^, 220 stings/min^32^, and 265 stings/min^46^. In contrast, our *scutellata*-hybrid colonies stung at a rate ~ 6 stings/min in the month they were least defensive (May) and ~ 66 stings/min in the month when they were the most defensive (October). Thus, the *scutellata*-hybrids assessed here are apparently less defensive than other *scutellata*-hybrids previously studied. However, these comparisons in stinging rate should be interpreted cautiously given methodological differences across studies. Some studies preceded their stinging assay with a disturbance while others did not. Of the studies reviewed in Table S6, half used colonies with 9–10 frames, while the rest used colonies with 3–4 frames. In addition, averaging the number of stings on a target over time does not capture potential variation in the rate of stinging during the observation periods, which varied between 20 and 60 s.

Lower defensiveness could be adaptive. The gentle *scutellata*-hybrids of Puerto Rico may have arisen from a combination of human intervention, geographic isolation, and a relative absence of predation^30,32,62^. Selection to maintain high defensiveness may have been relaxed because feral honey bees on this island do not face sufficient predation to compensate for the costs of intense nest defensiveness, which can result in more worker deaths. It is unknown if feral honey bees in Southern California face lower predation risk than feral honey bees in Central and South America. However, the high urbanization in Southern California might lead to greater extermination of highly defensive colonies, as was proposed in Puerto Rico. Given the high population of feral *scutellata*-hybrids in Southern California^41,42,63^ it is not clear if such extermination could significantly reduce average defensiveness of *scutellata*-hybrids, but this possibility is worth considering. Finally, relative gentleness in Southern California *scutellata-*hybrids might be evolving in response to distinct environmental factors in this region.

The lower amount of African ancestry (~ 40%) reported in the *scutellata*-hybrids of Southern California, approximately half the amount as that measured in Mexico and South America^43,49,58,64^ could explain the reduced hybrid defensiveness that we observed. However, F1 hybrids from the crossing of *scutellata*-hybrids of high African ancestry and EHB colonies in Mexico exhibited levels of defensive behavior similar to that seen in the parental *scutellata*-hybrid strain^18–25,24^. Thus, *scutellata*-hybrids with levels of *A. m. scutellata* genomic content similar to those measured in Southern California, can exhibit high defensiveness. However, the *scutellata*-hybrids measured here are likely not F1 hybrids (Fig. 2), and their decreased defensiveness could arise from multiple generations of hybridization.

*Scutellata*-hybrids showed reduced defensiveness in the earlier months and increased defensiveness later in the year (August–November). In San Diego, these fall months correspond to a time in which the number of native and non-native flowering plants decreases by over 50% in comparison with earlier spring and summer months^65^. At times of resource scarcity, bees may engage in robbing, which initiates a complex behavioral cascade in honey bee colonies—notably an increase in defensiveness by nest guards^39,40,66^. Brain gene expression profiles suggest that guards become unusually aggressive when their colony is robbing another or when they are being robbed^39^. Once robbing begins in an apiary, it may set off a cascade of robbing and aggression among multiple colonies^66^. Unlike pEHB colonies, which were fed during months of floral dearth, the *scutellata*-hybrids were never fed and were thus more likely to rob each other.

For the pEHB colonies at the BFS, colony size did not significantly affect defensive behavior, despite substantial variation in colony sizes across months at this site (Table 1 and Table S3). The changes in pEHB defensiveness over time depended upon the measure used. For three measures (stings on gloves, pursuit to 25 m, and pursuit to 50 m, defensiveness decreased over time. For one measure, flying of the comb, defensiveness increased over time. If we examine data from October, when our pEHB colonies stung flags at the highest rates, colonies averaged ~ 27 stings/min. Stinging rates for EHB measured in other studies are often below 30 stings/min: 6.3 stings/min^60^, 20 stings/min^25^, 22.8 stings/min^46^, 24.2 stings/min^20^, 24.7 stings/min^24^, 26.4 stings/min^18^, 24 stings/min^19^, 38 stings/min^21^, and 132 stings/min^23^. Thus, our pEHB had stinging rates similar to those reported for EHB in other studies.

Because we maintained pEHB colonies and *scutellata*-hybrid colonies in separate apiaries, changes in defensiveness and colony sizes may be due to multiple factors, including feeding pEHB colonies and treating them with medications to maintain their health. These practices are standard in most managed apiaries and are essential for reducing mortality of EHB colonies. An important question is therefore whether managed EHB and *scutellata*-hybrids vary over time in their defensiveness due to differences in management practices. Given the issues of robbing and interactive aggression effects between EHB and *scutellata*-hybrids discussed earlier, it was not possible for us to use a common garden design. Therefore, with respect to differences between pEHB and *scutellata*-hybrids, the trends shown in Fig. 1 should be considered cautiously because colony genetics and management practices covaried between the apiaries. However, if we consider studies conducted at multiple different locations in markedly different environments (and accounting for issues with calculating the average sting rate, see above), we find that *scutellata*-hybrids (AHB) sting an object at around 61–220 stings/min whereas EHB sting at about 20–38 stings/min (Table S6). Thus, the higher defensiveness of AHB as compared to EHB is largely consistent throughout the range of AHB.

In Southern California, nearly all freely foraging bees appear to be *scutellata*-hybrids. Zarate et al. sampled 15 freely foraging honey bee workers from widely separated sites in San Diego County and found that all had substantial *A. m. scutellata* ancestry (mean = 38%) with modest variation among individuals (25–46%)^43^. In regions such as Southern California with a high frequency of feral *scutellata*-hybrids, maintaining little or no introgression into managed stocks, as foreseen by Schneider et al*.* is challenging^13^. If the original queen dies or is superseded, the colony can rear new virgin queens that will mate with local drones, likely to be feral *scutellata*-hybrids. These matings could therefore result in workers with substantial amounts of *scutellata* ancestry. *Scutellata*-hybrid swarms can also take over existing EHB colonies—this was estimated to occur in 10–25% of Arizona colonies^13,67^. Thus, introgression through mating with feral drones, or penetration of *A. m. scutellata* ancestry into managed apiaries by usurpation, is difficult to entirely avoid.

When managed colonies become defensive, requeening with EHB queens is standard practice. However, requeening can be challenging. *Scutellata*-hybrid colonies reject requeening with EHB queens more often than EHB colonies^68^. Breeding practices can also complicate requeening. The pEHB colonies at our BFS apiary carried various amounts of *A. m. scutellata* ancestry (mean = 23%; range 7–38%). Although BFS colonies originated as nucleus colonies bred and reared outside the range of the *scutellata*-hybrids, when queens from nucleus colonies died, we requeened with Varroa Sensitive Hygiene *A. mellifera ligustica* queens bred for low defensiveness. These VSH queens were bred in Southern California, where they mated openly in a breeding area purposefully stocked with a high density of EHB colonies to favor insemination by EHB drones, but the local abundance of feral *scutellata*-hybrids may compromise such efforts.

S*cutellata*-hybrid colonies in Southern California apparently have relatively low levels of defensiveness compared to *scutellata*-hybrids in Mexico and South America. In addition, the ecological success of feral bees in Southern California^41–43^ suggests that their colonies thrive in the face of multiple health challenges for which managed hives require treatment. Geffre et al*.* showed that the infection levels of multiple viral honey bee pathogens, which are commonly spread by *Varroa* parasites, are similar for feral and managed honey bee foragers in Southern California, even though feral bees do not receive treatments against pathogens or parasites^69^. The ability of feral, *scutellata*-hybrid honey bees to thrive without supplemental feeding in periods of resource scarcity and to maintain colony strength without anti-pathogen treatments suggest that these feral bees may be a reservoir for desirable traits that can be potentially bred into commercial honey bees, possibly without significantly raising defensiveness.

### Supplementary Information

Supplementary Information.

## Data Availability

The DNA data used in this paper has been deposited on the Dryad database (https://datadryad.org/stash/share/-JGCfQNVQ_6pUI65SiE1SugeYOO75t-QwwBguVLp2Qw).
