# Supplementary material for: Seasonal variation in defense behavior in European and scutellata-hybrid honey bees (Apis mellifera) in Southern California
Source: Sci Rep. 2023 Aug 7;13:12790. doi: 10.1038/s41598-023-38153-2 (PMC10406949; doi:10.1038/s41598-023-38153-2)
Supplement: Supplementary file 1 — Supplementary Information. [file 41598_2023_38153_MOESM1_ESM.docx]

**SUPPLEMENTAL INFORMATION**

Accompanying submission of the manuscript “Seasonal variation in defense behavior in European and *scutellata*-hybrid honey bees (*Apis mellifera*) in Southern California” by Zarate et al. to *Scientific Reports.*


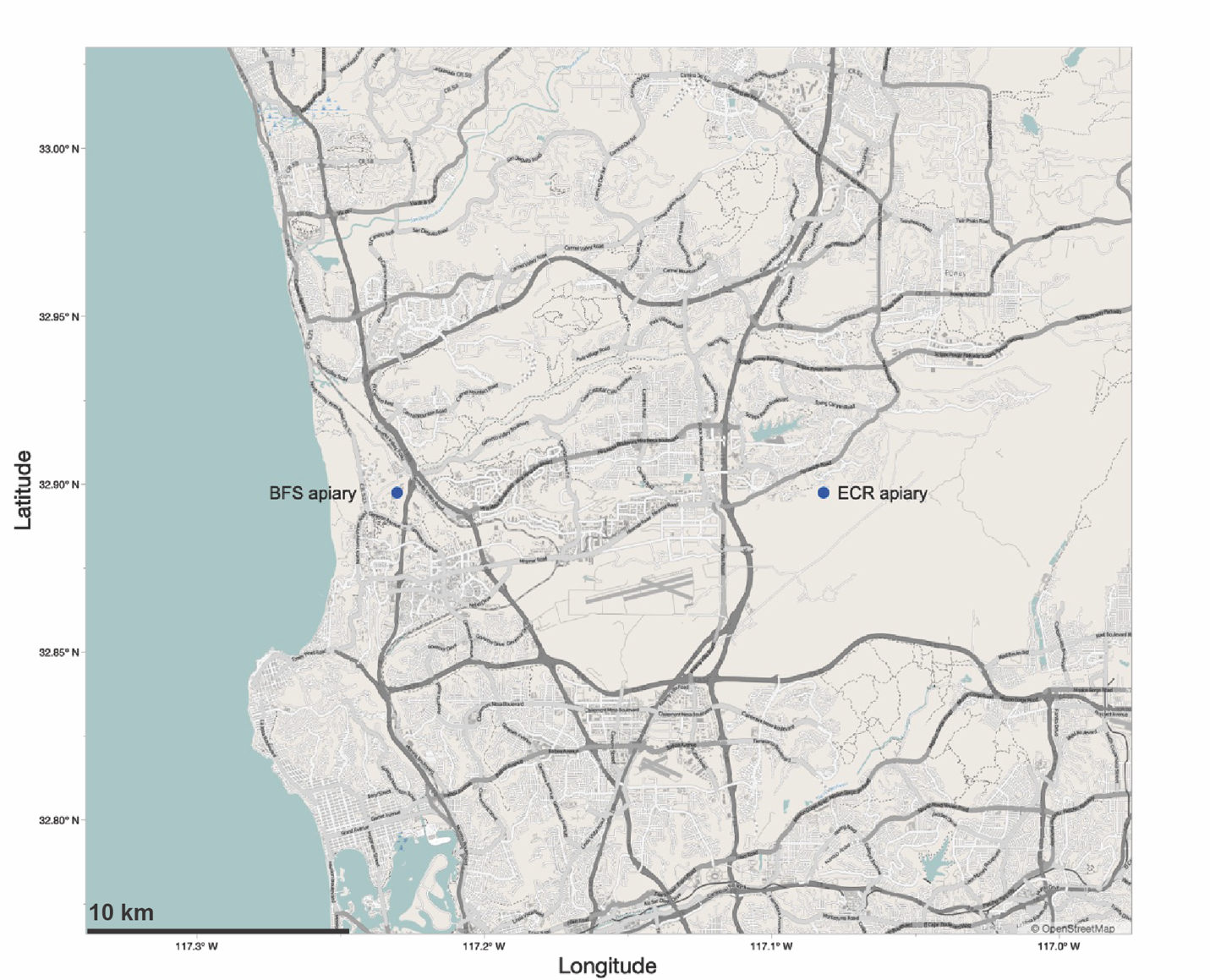


**Figure S1**: Map of San Diego County, California, USA where this study occurred, blue dots mark the BFS and ECR apiary. Figure produced with JMP v16.1, SAS Institute Inc, Cary, NC.

**Table S1:** Rubric for qualitatively scoring honey bee defensiveness.

| Defense Metric | 1  (Low) | 2 | 3 | 4 | 5  (High) |
| --- | --- | --- | --- | --- | --- |
| Fly Off Comb  (# of bees flying around the box from moment of opening to closing of the box) | 0-10 | 10-20 | 20-30 | 30-40 | 50+ |
| Hit Operator’s Veil  (# of bees touching or mobbing veil from moment of opening to closing of the colony box) | 0-5 | 5-10 | 10-15 | 15-30 | 30+ |
| Pursuit to 25 meters  (# of bees flying around operator at the 25 m mark) | 0-5 | 5-10 | 10-15 | 15-30 | 30+ |
| Pursuit to 50 meters  (# of bees flying around operator at the 50 m mark) | 0-5 | 5-10 | 10-15 | 15-30 | 30+ |

**Table S2**. Repeated measured mixed-effects model assessing the effects of sampling order on each defensive measure with each site analyzed separately. We included time, and order of testing as fixed effects, colony as a random effect and any significant interactions between main effects in the model. Colony and any interactions containing colony were designated as random effects. Stings on gloves were log transformed to improve model residuals. We report the fixed effect tests of order as the source here. Order does not emerge as a significant predictor of any defensive measure at either site (p < 0.0253).

| Site | Defensive Behavior | Nparm | DF | DFDen | F Ratio | Prob > F |
| --- | --- | --- | --- | --- | --- | --- |
| BFS | Stings on Flag | 1 | 1 | 7.77 | 0.309 | 0.594 |
|  | Stings on Gloves | 1 | 1 | 25.7 | 2.34 | 0.138 |
|  | Fly off Comb | 1 | 1 | 7.51 | 3.31 | 0.109 |
|  | Hit Veil | 1 | 1 | 31.8 | 2.09 | 0.158 |
|  | Pursuit to 25 meters | 1 | 1 | 29.6 | 1.24 | 0.275 |
|  | Pursuit to 50 meters | 1 | 1 | 28.2 | 1.42 | 0.243 |
| ECR | Stings on Flag | 1 | 1 | 8.96 | 0.036 | 0.854 |
|  | Stings on Gloves | 1 | 1 | 7.58 | 0.070 | 0.799 |
|  | Fly off Comb | 1 | 1 | 20.8 | 0.013 | 0.911 |
|  | Hit Veil | 1 | 1 | 32.3 | 0.085 | 0.773 |
|  | Pursuit to 25 meters | 1 | 1 | 13.34 | 0.077 | 0.786 |
|  | Pursuit to 50 meters | 1 | 1 | 22.6 | 0.547 | 0.467 |

**Table S3**. Average colony sizes at ECR and BFS between May and November. We were unable to collect colony size data during the month of July.

| Month | Average BFS Colony Size | Average ECR Colony Size |
| --- | --- | --- |
| May | 8476 ±1381 | 4522 ± 1151 |
| July | - | - |
| August | 3572 ± 903 | 5661± 952 |
| October | 5920 ± 941 | 5770 ± 1411 |
| November | 4978 ± 881 | 3954 ± 1015 |

**Table S4**. The effect of colony size on the six measures of defensiveness that were analyzed jointly in a multivariate analysis of variance (MANOVA) within each site and calendar month. We excluded the month of July for both sites as we lacked colony size data for this month. We also excluded the ECR site in the month of October because we had too few colony size measurements to perform this analysis.

| Site | Month | Value | Exact F | NumDF | DenDF | Prob>F |
| --- | --- | --- | --- | --- | --- | --- |
| BFS | May | 4.15 | 2.08 | 6 | 3 | 0.293 |
|  | July | - | - | - | - | - |
|  | August | 1.78 | 0.89 | 6 | 3 | 0.589 |
|  | October | 4.34 | 2.17 | 6 | 3 | 0.280 |
|  | November | 1.58 | 0.79 | 6 | 3 | 0.633 |
| ECR | May | 2.82 | 1.41 | 6 | 3 | 0.419 |
|  | July | - | - | - | - | - |
|  | August | 4.67 | 2.34 | 6 | 3 | 0.260 |
|  | October | - | - | - | - | - |
|  | November | 6.60 | 3.30 | 6 | 3 | 0.177 |

**Table S5**. Variation in amount of *A. m. scutellata* ancestry as explained by colony identity analyzed separately for both ECR and BFS apiary with an analysis of variance (ANOVA).

| Site | Source | Nparm | DF | Sum of Squares | F Ratio | Prob > F |
| --- | --- | --- | --- | --- | --- | --- |
| BFS | Colony | 9 | 9 | 0.230 | 5.89 | 0.0005 |
| ECR | Colony | 9 | 9 | 0.016 | 6.99 | 0.0002 |

**Table S6**. Summary of studies measuring defensive behavior in *scutellata*-hybrids (also known as Africanized honey bees or AHB) or European honey bees (EHB) using assays that count the number of stings in an object over time. EHB in these studies were sourced from USA, Canada, or from established lineages of managed honey bees of known European lineage.

| Citation | Location | Target of Stings and time measured (s) | Honey bee Genotype | Colony average sting rate ± SEM  (n=colonies) | | | Effect size (Cohen’s *d*) | Additional Notes |
| --- | --- | --- | --- | --- | --- | --- | --- | --- |
| Guzman-Novoa & Page, Jr. (1993) | Tonatico, Mexico | Black leather flag (60 s); colonies tested three times (twice on the same day, once 2 days later) | AHB* | 110.3 ± 4.49  (n=3) (a) | 129.7 ± 5.89  (n=3) (d) | 146.7 ± 7.60 (n=3) (f) | 14.3 (averaged between a-b. c-d, and e-f comparisons) | *The same three colonies were used in each of these measures for AHB and EHB.  a-f: Genotypes sharing the same letter do not differ significantly from each other. Comparisons valid only between a-b, c-d, and e-f (P < 0.001).  10 frames used (4 of capped brood, 6 frames covered with adult bees). |
|  |  |  | EHB* | 23.3 ± 1.39  (n=3) (b) | 27.0 ± 3.01  (n=3) (c) | 22.3 ± 3.27  (n=3) (e) |  |  |
|  |  |  | F1 Hybrids  (AHB ♀ x EHB ♂) | 92.8 ±14.23 (n=10) (a) |  |  |  |  |
|  |  |  | Backcross 1  (F1 ♀ x EHB ♂) | 42.3 ± 7.81 (n=11) (c) |  |  |  |  |
|  |  |  | Backcross 2  (F1 ♀ x EHB ♂) | 19.4 ± 3.88  (n=10) (e) |  |  |  |  |
| Guzman-Novoa *et al.,* (2004) | Villa Guerrero, Mexico | Black leather flag (20 s); colonies tested 8x each, once per day | AHB | 98 (n=1) | 45 (n=1) | 45 (n=1) | Insufficient data provided to calculate effect size (standard deviation or standard error not reported) | Averages and SEM not reported, statistical analysis between AHB and EHB not conducted  4 frames used (2 frames of brood, 2 frames of pollen + nectar). |
|  |  |  | EHB | 44 (n=1) | 0 (n=1) | 0 (n=1) |  |  |
| Stort, 1974 | Ribeirao Preto (SP), Brazil | Black leather ball (60 s); colonies tested 5x each in 10 min intervals | AHB | 61.2 ± 14.3 (n=9) |  |  | 1.02 | AHB and EHB significantly different (P < 0.01).  Colonies composed of a nucleus with 3 frames of brood. |
|  |  |  | EHB | 26.4 ± 10.0 (n=5) |  |  |  |  |
| Villa 1988 | Colombia | Black leather flag (30 s); colonies tested 1x at both high (2450 m) and low (520 m) elevation | AHB | 31.5 ± 5.95 (n=10) § | 15.2 ± 3.94 (n=10) ° |  | 1.58§  1.74° | § Colonies tested at high elevation  ° Colonies tested at low elevation  AHB and EHB significantly different (P < 0.01).  Colonies were composed of 4 frames covered with adult bees. |
|  |  |  | EHB | 5.1 ± 1.87 (n=8) § | 1.2 ± 0.49 (n=8) ° |  |  |  |
| Guzman-Novoa *et al.,* (2002) | Ixtapan de la Sal, Mexico | Black leather flag (60 s); preceded by initial visual stimulus of waving flag; colonies tested 3x each on the same day | AHB | 125 (n=12) (c) |  |  | Insufficient data provided to calculate effect size (standard deviation or standard error not reported) | a-c: Genotypes sharing the same letter do not differ significantly from each other (P < 0.0001). Actual values visually estimated from Fig. 1. SEM not explicitly provided.  F1 Hybrids were both AHB ♂ x EHB ♀ and AHB ♀ x EHB ♂.  Colonies were composed of 4 frames of capped brood and 5 frames of adult bees. |
|  |  |  | EHB | 38 (n=9) (a) |  |  |  |  |
|  |  |  | F1 Hybrid | 115 (n=9) (bc) |  |  |  |  |
|  |  |  | AHB Backcross  (F1 ♀ x AHB ♀) | 136 (n=19) (c) |  |  |  |  |
|  |  |  | EHB Backcross  (F1 ♀ x EHB ♂) | 100 (n=21) (b) |  |  |  |  |
| Collins *et al.,* 1982 | Monagas, Venezuela (AHB)  Louisiana, USA (EHB) | Black leather flag (30 s); colonies tested 2x in 1 day | AHB | 82 (n=150)† | 14 (n=15)‡ |  | Insufficient data provided to calculate effect size (standard deviation or standard error not reported) | AHB and EHB significantly different in both experimental designs (P < 0.01). SEM or SD not explicitly provided.  † More colonies, but assessed separately in a split-apiary design: AHB tested in Venezuela, EHB tested in Louisiana  ‡ Fewer colonies in a common garden apiary design in Venezuela.  Colonies consisted of 3 frames. |
|  |  |  | EHB | 12 (n=147)† | 2 (n=15)‡ |  |  |  |
| Rivera-Marchand *et al,* (2012) | Puerto Rico | Black leather flag (60 s); Assay preceded by impact to nest box; colonies tested 2x with a week interval | AHB | 220 (SD: 142) (n=9)^1^ (a) |  |  | 0.131 | a: Genotypes sharing the same letter do not differ significantly from each other (P > 0.5).  SEM not reported, SD given instead.  Number of frames per colony were not reported. |
|  |  | Black leather flag (60 s); colonies tested 2x with a 2-day interval | EHB (Reported from Giray et al. 2000) | (197.1 SD:201.4) (n=13)^1^ (a) |  |  |  |  |
| Guzman-Novoa & Page, Jr. (1994) | Ixtapan de la Sal, Mexico | Black leather flag (60 s); colonies tested three times (twice on the same day, once 2 days later) | AHB | 137.3 (n=5) (b) | 153.0 ± 9.91 (n=3) (b)* |  | Insufficient data provided to calculate effect size (standard deviation or standard error not reported for most measures) | F1 hybrid mixtures created from inseminating an EHB ♂ with AHB ♀ with varying semen proportions.  a-b: Genotypes sharing the same letter do not differ significantly from each other (P < 0.001). Some values visually estimated from Fig. 1.  *Three randomly picked AHB colonies from the first five assayed were tested again two weeks after initial tests.  Colonies were composed of 3-4 frames of capped brood and 6 frames of adult bees. |
|  |  |  | EHB | 24.7 (n=8) (a) |  |  |  |  |
|  |  |  | 14% F1 Hybrid | 42.0 (25% hybrid) (a) |  |  |  |  |
|  |  |  | 30 % F1 Hybrid | 84.3 (50% hybrid) (b) |  |  |  |  |
|  |  |  | 50% F1 Hybrid | 130 (b) |  |  |  |  |
| Guzman-Novoa *et al.,* (2005) | Ixtapan de la Sal, Mexico | Black leather flag (60 s); assay preceded with visual stimulus; colonies tested 3-8x; | AHB | 110 (n=32) |  |  | Insufficient data provided to calculate effect size (standard deviation or standard error not reported for most measures) | Values are summary counts of the number of stings for four genotypes over four years of testing. Statistical tests were not performed on this averaged data and SEM not given. Values were estimated from Fig. 1.  Colonies were composed of 4 frames of capped brood and 5 frames of adult bees. |
|  |  |  | EHB | 20 (n=31) |  |  |  |  |
|  |  |  | F1 Hybrid (AHB ♀ x EHB ♂) | 65 (n=28) |  |  |  |  |
|  |  |  | F1 Hybrid (AHB ♂ x EHB ♀) | 125 (n=124) |  |  |  |  |
| Guzman-Novoa *et al.,* (2003) | Villa Guerrero, Mexico | Black leather flag (20 s); assay preceded with visual stimulus | AHB | 88.3 ± 14.1 (n=6) (a) |  |  | 2.37 | a-b: Genotypes sharing the same letter do not differ significantly from each other (P < 0.001).  F1 Hybrids were both AHB ♂ x EHB ♀ and AHB ♀ x EHB ♂  Colonies contained 7 frames of adults bees and 6000 cm^2^ od capped brood. |
|  |  |  | EHB | 7.6 ± 6.5 (n=6) (b) |  |  |  |  |
|  |  |  | F1 Hybrid | 77.5 ± 23.9 (n=6) (a) |  |  |  |  |

**SI REFERENCES**

Guzmán-Novoa, E. & Page, R. E. Backcrossing Africanized honey bee queens to European drones reduces colony defensive behavior. *Ann. Entomol. Soc. Am.* **86**, 352-55 (1993).

Guzmán-Novoa, E., Hunt, G. J., Uribe-Rubio, D. & Prieto-Merlos, D. Genotypic effects of honey bee (*Apis mellifera*) defensive behavior at the individual and colony levels: the relationship of guarding, pursuing, and stinging. *Apidologie*, **35**, 15-24, (2004).

Stort, C. Genetic Study of Aggressiveness of Two Subspecies of *Apis mellifera* in Brazil 1. Some Tests to Measure Aggressiveness. *Journal of Apicultural Research.* **13**(1), 33-38 (1974).

Villa, J. D. Defensive Behavior of Africanized and European Honeybees at Two Elevations in Colombia. *Journal of Apicultural Research*, **27**(3), 141-145, (1988).

Guzmán-Novoa, E., Hunt, G. J., Uribe, J. L., Smith, C. Arechavaleta-Velasco, M. E. Confirmation of QTL effects and evidence of genetic dominance of honeybee defensive behavior: Results of colony and individual behavioral assays. *Behavior Genetics*, **32**(2), 95-102, (2002).

Collins, A. M. Rinderer, T. E., Harbo, J. R. & Bolten, A. B. Defense by Africanized and European Honey Bees. *Science*, **218**(4567), 72-74, (1982).

Rivera-Marchand, B., Oskay, D,, & Giray, T. Gentle Africanized bees on an oceanic island. *Evolutionary Applications*, **5**(7), 746-56, (2012).

Guzmán-Novoa, E. & Page, R. E. Genetic dominance and worker interactions affect honeybee colony defense. *Behavioral Ecology*, **5**(1), 91-97, (1994).

Guzmán-Novoa E., Hunt G. J., Page Jr. R. E., Uribe-Rubio D., Prieto-Merlos, D. & Becerra-Guzman, F. Paternal effects on the defensive behavior of honeybees. *J. Heredity,* **96**(4), 376-380, (2005).

Guzmán-Novoa, E., Prieto-Merlos, D., Uribe-Rubio, J. L. & Hunt, G. J. Relative reliability of four field assays to test defensive behaviour of honey bees (*Apis mellifera*). *Journal of Apicultural Research*. **42**(3), 42-46 (2003).
